# P-771. Demographic and Clinical Characteristics of Adverse Reactions to anti-Tuberculosis Therapy: A Retrospective Analysis

**DOI:** 10.1093/ofid/ofae631.966

**Published:** 2025-01-29

**Authors:** Drieda Zaçe, Rossi Benedetta, Maria Letizia Minardi, Grazia Alessio, Leonardo Alborghetti, Gianmarco Muratore, Martina Moccione, Luigi Coppola, Mirko Compagno, Laura Campogiani, Iannetta Marco, Loredana Sarmati

**Affiliations:** University of Rome Tor Vergata, Rome, Lazio, Italy; Tor Vergata Hospital, Rome, Lazio, Italy; 1- Department of Systems Medicine, Infectious Disease Clinic, Tor Vergata University, Rome, Lazio, Italy; Tor Vergata Hospital, Rome, Lazio, Italy; 1- Department of Systems Medicine, Infectious Disease Clinic, Tor Vergata University, Rome, Lazio, Italy; 1- Department of Systems Medicine, Infectious Disease Clinic, Tor Vergata University, Rome, Lazio, Italy; 1- Department of Systems Medicine, Infectious Disease Clinic, Tor Vergata University, Rome, Lazio, Italy; Tor Vergata Hospital, Rome, Lazio, Italy; Tor Vergata Hospital, Rome, Lazio, Italy; Tor Vergata University of Rome, Rome, Lazio, Italy; University of Rome Tor Vergata , Rome, Lazio, Italy; Tor Vergata University, Rome, Rome, Lazio, Italy

## Abstract

**Background:**

Adverse reactions (AR) to anti-tuberculosis (anti-TB) therapy pose a significant challenge, leading to decreased treatment compliance, increased morbidity and mortality rates, and prolonged hospital length-of-stay.

**Figure:**
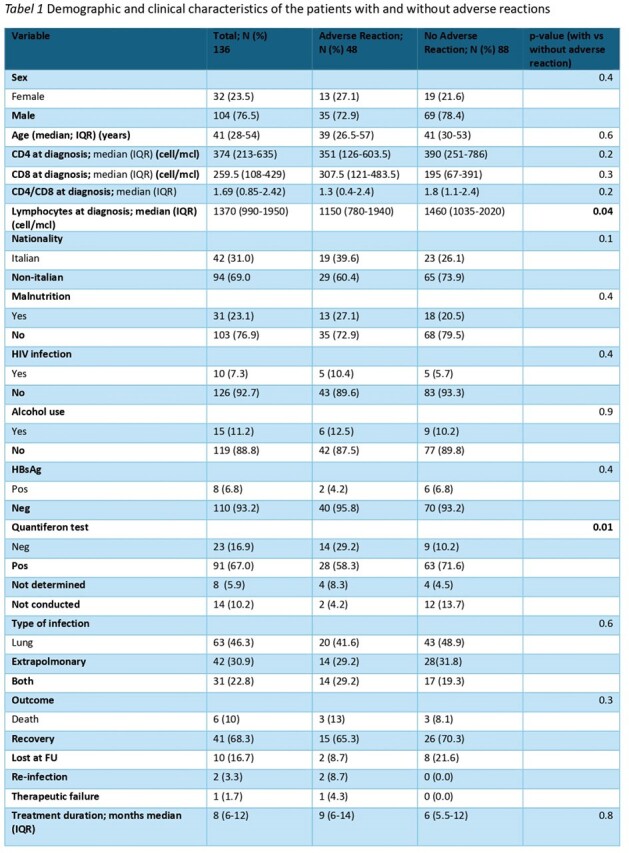

**Methods:**

This study examines the demographic, clinical, and laboratory profiles of patients diagnosed with tuberculosis and hospitalized at the Tor Vergata Policlinic in Rome between 2013-2023, who experienced adverse reactions requiring the interruption, suspension, or modification of anti-tuberculosis therapy, in the period 2013-2023.

**Results:**

Out of 242 patients receiving treatment for tuberculosis, data regarding AR were available for 136 cases. Of these, 35.3% (48/136) experienced an AR attributable to anti-tuberculosis therapy, median age of 39 years (IQR 26.5-57), 72.9% male, 60.4% non-italians, and 41.6% undergoing treatment for pulmonary TB. The most common AR were hypertransaminasemia (52.2%), allergic reaction (25%), and cholestasis (8.3%).

Patients with AR characterized by hypertransaminasemia/cholestasis compared to those with allergic reactions were younger (31 years (IQR 25-45) vs. 60.5 (IQR 36-69.5); p=0.012), more frequently of non-italian nationality (74.2% vs. 25.8%; p= 0.013), and had more frequently a negative Quantiferon test (41.9% vs. 8.3%; p= 0.03).

Pirazinamide emerged as the drug most frequently linked to AR, being suspended in 62.5% of cases (20/29 due to hypertransaminasemia; 8/12 due to allergic reaction). In 76.2% of cases, the drug responsible for the AR was discontinued in favor of a fluoroquinolone. All ARs resolved upon suspension of the implicated drug.

Among patients with AR, 65.3% recovered, and 13% died. Patients with AR had a lower lymphocyte count at treatment initiation compared to patients without AR (median 1150 (IQR 780-1940) vs 1460 (IQR 1035-2020), p=0.04) and had more frequently a negative Quantiferon test (29.2% vs 10.2%; p=0.012).

**Conclusion:**

AR during TB therapy affected 35% of patients in our sample. Pyrazinamide was the most commonly implicated drug, and the reactions resolved upon discontinuation. A lower lymphocyte count at TB diagnosis in patients with AR might indicate an immune component, similar to IRIS, in their onset. However, further studies are warranted to explore this aspect.

**Disclosures:**

**All Authors**: No reported disclosures

